# Effectiveness of a Very Early Stepping Verticalization Protocol in Severe Acquired Brain Injured Patients: A Randomized Pilot Study in ICU

**DOI:** 10.1371/journal.pone.0158030

**Published:** 2016-07-22

**Authors:** Giuseppe Frazzitta, Ilaria Zivi, Roberto Valsecchi, Sara Bonini, Sara Maffia, Katia Molatore, Luca Sebastianelli, Alessio Zarucchi, Diana Matteri, Giuseppe Ercoli, Roberto Maestri, Leopold Saltuari

**Affiliations:** 1 Department of Brain Injury and Parkinson Disease Rehabilitation, Ospedale “Moriggia-Pelascini”, Gravedona ed Uniti (CO), Italy; 2 Department of Intensive Care, Ospedale “Moriggia-Pelascini”, Gravedona ed Uniti (CO), Italy; 3 Department of Biomedical Engineering, Scientific Institute of Montescano, Fondazione S. Maugeri IRCCS, Montescano (PV), Italy; 4 Research Unit for Neurorehabilitation South Tyrol, Landeskrankenhaus Hochzirl-Natters, Zirl, Austria; University of Glasgow, UNITED KINGDOM

## Abstract

**Background and Objective:**

Verticalization was reported to improve the level of arousal and awareness in patients with severe acquired brain injury (ABI) and to be safe in ICU. We evaluated the effectiveness of a very early stepping verticalization protocol on their functional and neurological outcome.

**Methods:**

Consecutive patients with Vegetative State or Minimally Conscious State were enrolled in ICU on the third day after an ABI. They were randomized to undergo conventional physiotherapy alone or associated to fifteen 30-minute sessions of verticalization, using a tilt table with robotic stepping device. Once stabilized, patients were transferred to our Neurorehabilitation unit for an individualized treatment. Outcome measures (Glasgow Coma Scale, Coma Recovery Scale revised -CRSr-, Disability Rating Scale–DRS- and Levels of Cognitive Functioning) were assessed on the third day from the injury (T0), at ICU discharge (T1) and at Rehab discharge (T2). Between- and within-group comparisons were performed by the Mann-Whitney U test and Wilcoxon signed-rank test, respectively.

**Results:**

Of the 40 patients enrolled, 31 completed the study without adverse events (15 in the verticalization group and 16 in the conventional physiotherapy). Early verticalization started 12.4±7.3 (mean±SD) days after ABI. The length of stay in ICU was longer for the verticalization group (38.8 ± 15.7 vs 25.1 ± 11.2 days, p = 0.01), while the total length of stay (ICU+Neurorehabilitation) was not significantly different (153.2 ± 59.6 vs 134.0 ± 61.0 days, p = 0.41). All outcome measures significantly improved in both groups after the overall period (T2 vs T0, p<0.001 all), as well as after ICU stay (T1 vs T0, p<0.004 all) and after Neurorehabilitation (T2 vs T1, p<0.004 all). The improvement was significantly better in the experimental group for CRSr (T2-T0 p = 0.033, T1-T0 p = 0.006) and (borderline) for DRS (T2-T0 p = 0.040, T1-T0 p = 0.058).

**Conclusions:**

A stepping verticalization protocol, started since the acute stages, improves the short-term and long-term functional and neurological outcome of ABI patients.

**Trial Registration:**

clinicaltrials.gov NCT02828371

## Introduction

Acquired brain injuries (ABIs) result from traumatic and non-traumatic (mostly hemorrhagic, hypoxic, ischemic, infective and toxic) cerebral events and may lead to coma state in the acute phase. The most severe patients often do not achieve a complete recovery of consciousness, evolving in a Vegetative State (VS) or in a Minimally Conscious State (MCS) [1–2]. The prevalence of disorders of consciousness (DOC) is around 0.2–6.1/100000 inhabitants [3]. Considering the increasing number of survivors, the long and expensive hospitalization and the remaining functional disabilities, ABIs represent a relevant clinical and social problem [4–5].

International guidelines support the indication to hospitalize brain injured patients in neurological intensive care units (NeuroICU), in order to better manage primary and secondary mechanisms of damage [6–7]. Furthermore, several authors emphasize the value of an integrated approach, in which both the acute care and the rehabilitative treatment are carried out at the same time under one interdisciplinary team [8–11]. Indeed, increasing evidence show that starting rehabilitation in NeuroICU is safe and feasible, helps to improve patients’ functional outcome and leads to a shorter hospitalization with lower costs [10,12,13]. An early generic mobilization by a physiotherapist improves circulation, ventilation and muscle metabolism, leading to a reduction of physical deconditioning, ventilator dependence and risk of complications (e.g. bed rest syndrome, infections, pressure ulcers, osteoporosis, deep vein thrombosis) and to an improvement of arousal, functional communication and psychological profile [14–18]. However, because of the lack of widely approved protocols, how and when patients with ABI should be mobilized out of bed is still controversial. Recently, the AVERT (A Very Early Rehabilitation Trial) investigators stated that a very early in-bed and out-of-bed mobilization of ischemic and hemorrhagic stroke patients in Stroke Unit leads to a less favorable outcome. However, their very early rehabilitation was carried out in the first 24 hours from the event (in the acute unstable phase of the brain injury) and only five hours before the physiotherapy timing of the controls [19].

Regarding DOC patients, one of the most important component of their mobilization is the positional change, possible by placing the patient on a tilt table. Verticalization stimulates several sensorial pathways and postural reactions, improves the level of arousal and awareness and is safe even if started in ICU [20–23].

The use of tilt table in patients with ABI was unfortunately often limited (and thus the verticalization delayed) by the occurrence of orthostatic hypotension and syncope, due to blood pooling in the lower limbs [21,24]. The breakthrough was when in 2004 Czell et al demonstrated how, in normal subjects, a greater hemodynamic stability during tilt table could be achieved by applying passive stepping or cycling movements to the legs [25]. In this field of research, Luther and coll. studied the effects of a tilt table with an integrated robotic stepping device in patients with VS or MCS after >30 days from the brain injury. The authors showed that, compared to a conventional tilt table, patients treated with this robotic-stepping tilt table experienced syncope episodes with a lower incidence [24].

In a recent paper, our group demonstrated the feasibility and safety of the very early use of a tilt table with stepping device in NeuroICU patients with DOC due to severe traumatic brain injury (TBI). Patients started the stepping verticalization protocol (30-minute daily sessions, five days per week for three consecutive weeks) at 12.7±8.7 days from the event; none of them experienced either dangerous hemodynamics variations or adverse events [26]. Same results were obtained in patients with DOC after cerebrovascular accident (personal data).

Aim of the present study is to examine if a very early stepping verticalization protocol, compared with conventional in-bed mobilization in ICU, leads to a greater functional and neurological improvement in patients with VS and MCS after severe ABI.

## Methods

### Study design and setting

This is a parallel-group, single-center, single-blind randomized clinical trial, carried out in the Intensive Care Unit of ‘Moriggia-Pelascini’ Hospital, Gravedona ed Uniti (CO), Italy, by a multidisciplinary team composed of Anesthesiologists, Neurologists, a Physiatrist, Physiotherapists and Nurses. The study was originally approved as safety and feasibility study by the local research Ethics Committee on October 11^th^ 2012. Upon approval from our Institutional Review Board, it was converted to a pilot randomized clinical trial. The trial was retrospectively registered on clinicaltrials.gov (NCT02828371), being not mandatory before recruitment. A written informed consent was obtained from the next of kin for each patient in the study. The individuals in this manuscript have given written informed consent to publish these case details. The study protocol is available as supporting information file (S1 Protocol). The authors confirm that all ongoing and related trials for this drug/intervention are registered.

### Subjects

We assessed consecutive patients with DOC admitted from January 2013 to April 2015 to our NeuroICU within 24 hours from a severe ABI. Patients were identified by the ICU physicians and notified to the neurologists of the Neurorehabilitation department for evaluation of the enrollment criteria. Inclusion criteria were age ≥18 years; Glasgow Coma Scale (GCS) ≤8 for ≥24h from the event; diagnosis of VS or MCS, according to the Coma Recovery Scale revised (CRSr) [27] on the third day after the injury; adequate pulmonary gas exchanging function (arterial O2 pressure/O2 flux ratio ≥250); stable hemodynamics (absence of dangerous variations of Mean Arterial Pressure or Heart Rate), even if obtained with continuative amines support. Exclusion criteria: sedation; unstable intracranial pressure (ICP); cerebral perfusion pressure (CPP) <60 mmHg; fractures or skin lesions in thorax, abdomen or lower limbs; deep vein thrombosis; body weight >130 kg; height >210 cm. Intubation and mechanical ventilation were not considered barriers for the treatment.

### Randomization and masking

Patients were randomly assigned to the early stepping verticalization protocol or to the control group using a web-based application for block randomization (www.randomization.com). We used the block randomization method (block size = 4) in order to ensure balance in sample size across groups over time. The randomization procedure was ran by a single investigator not involved in the clinical management, working in an Institution independent from the enrolling Center. Only after the patient identifiers had been communicated, the individual patient allocation was revealed to the enrolling staff.

The outcomes assessor was blinded to treatment allocation and to the study design, while the rest of the personnel involved in the study (physiotherapists, nurses, physicians) could not.

### Procedures

Experimental group started the stepping verticalization protocol between the third and the 30th day after the ABI, only when a hemodynamic, respiratory and intracranial stability was achieved (see inclusion/exclusion criteria). The protocol consisted of single daily sessions of verticalization, using a tilt table with an integrated robotic stepping device (Erigo. Hocoma AG, Switzerland) located in the ICU room. The upper body of the patient was secured to the table by fastening the chest and the shoulders with a harness. The feet were strapped to the two footplates and the distal thighs secured to the stepping device. Legs stepping movements were passively obtained with the rhythmic alternating pushing up of the feet, and controlled by a computer [25]. After patient positioning, the slope of the tilt table was gradually increased from 0° to 20°, 40° and then 60° in a time span of nine minutes. The stepping frequency was set at 20 steps/min for the entire treatment. Cardiovascular and respiratory parameters were continuously monitored. The net time of the session was 30 minutes, excluding the time required to transfer the patient and set the machine. Sessions were performed with the supervision of a physiotherapist; an ICU nurse was always present in the room, while the intensive care physician was available in case of emergency (S1 Video). Sessions were performed five times per week (Monday-Friday) for three consecutive weeks (a total of 15 sessions per patient) [26]. On the same days the patients received conventional physiotherapy for 30 minutes a day. Before the verticalization period the experimental group received conventional in-bed physiotherapy for 60 minutes a day.

Four conditions were defined as criteria for verticalization session interruption: mean arterial pressure ≤70 mmHg, heart rate ≤40 or ≥150 bpm, oxygen saturation ≤90% and traumatic dislodgement of a device (tracheal cannula, venous or arterial catheter, bladder catheter). Changes in inclusion/exclusion criteria during the course of the treatment were criteria for treatment interruption. More severe adverse events, as neurological worsening or myocardial infarction, were conditions determining withdrawal from the study.

Controls were treated with conventional in-bed physiotherapy (mobilization exercises in supine and sitting position on bed, without out-of-bed mobilization nor verticalization) for 60 minutes a day, from Monday to Friday, throughout the ICU stay.

Patients of both groups received an individualized best medical therapy depending on the evolution of their comorbidities throughout their hospitalization and started brain stimulating drugs only after Neurorehabilitation admission.

Patients of both groups were moved from ICU to our Neurorehabilitation clinic once they reached a general and neurological clinical stability; in addition, patients of the experimental group could be moved only after completion of the stepping verticalization protocol. During their Neurorehabilitation stay, patients of both groups received specialized nursing care and an individualized rehabilitative treatment composed of conventional physiotherapy, robotics (including stepping verticalization sessions), devices weaning, speech/swallowing therapy, cognitive therapy and the best medical treatment, without differences between groups. Stepping verticalization sessions with Erigo during the Neurorehabilitation phase were administered to patients of both groups 3 times/week until they achieved the ability to move to a more complex robotic treatment. The length of stay in our Neurorehabilitation department for DOC patients is set at a maximum of 6 months. Hence, the last discharge from Neurorehabilitation was in November 2015.

### Outcome measures

We collected data about age, sex, etiology of the ABI, vascular risk factors and site of the main brain damage. As outcome measures we choose the following scales: GCS, Disability Rating Scale (DRS), CRSr and Levels of Cognitive Functioning (LCF). The scores for each scale were assessed from a blinded investigator on the third day from the injury (T0), at ICU discharge (T1) and at Rehabilitation discharge (T2). We then calculated for each score the following differences: T1-T0, T2-T1 and T2-T0, in order to evaluate the size of the neurological improvement for each patient and to define the short-term (at T1) and long-term (at T2) outcomes in the two groups.

### Statistical analysis

Sample size computation was not feasible since neither measures of standard error measurement (SEM) nor measures of the Minimally Clinically Important Difference were available for the outcome measures of our study [ref: http://www.rehabmeasures.org]. A sample size of convenience (20+20 patients) was therefore used as the study was regarded as a pilot trial.

Shapiro–Wilk statistic, supported by visual inspection, was used to assess the normality of the distribution of continuous variables.

Descriptive statistics for normally and non-normally distributed data are reported as mean±SD or median (lower quartile, upper quartile) respectively. Descriptive statistics for discrete variables are reported as number (percentage frequency).

The outcome measures violated the normality assumption. Accordingly, between- and within-group comparisons were performed by the Mann-Whitney U test and Wilcoxon signed-rank test, respectively. The independent samples t-test was used for between group comparison of clinical and demographic variables showing normal distribution. Comparisons of categorical variables were carried out with the Chi-square test or Fisher exact test when appropriate.

To assess whether an early stepping verticalization protocol may lead to a better improvement as compared to conventional mobilization in ICU, we first computed the difference (end of treatment-start of treatment) for all outcome variables and then compared these differences between the two groups by Mann-Whitney U test.

All statistical tests were two-tailed and statistical significance was set at p < 0.05. When appropriate, false discovery rate was controlled at 5% using the Benjamini-Hochberg method. Raw p values were reported with the information whether they were or not significant using the Benjamini-Hochberg procedure at a 5% false discovery rate.

All analyses were carried out using the SAS/STAT statistical package, release 9.2 (SAS Institute Inc., Cary, NC, U.S.A.).

## Results

**Fig 1:**
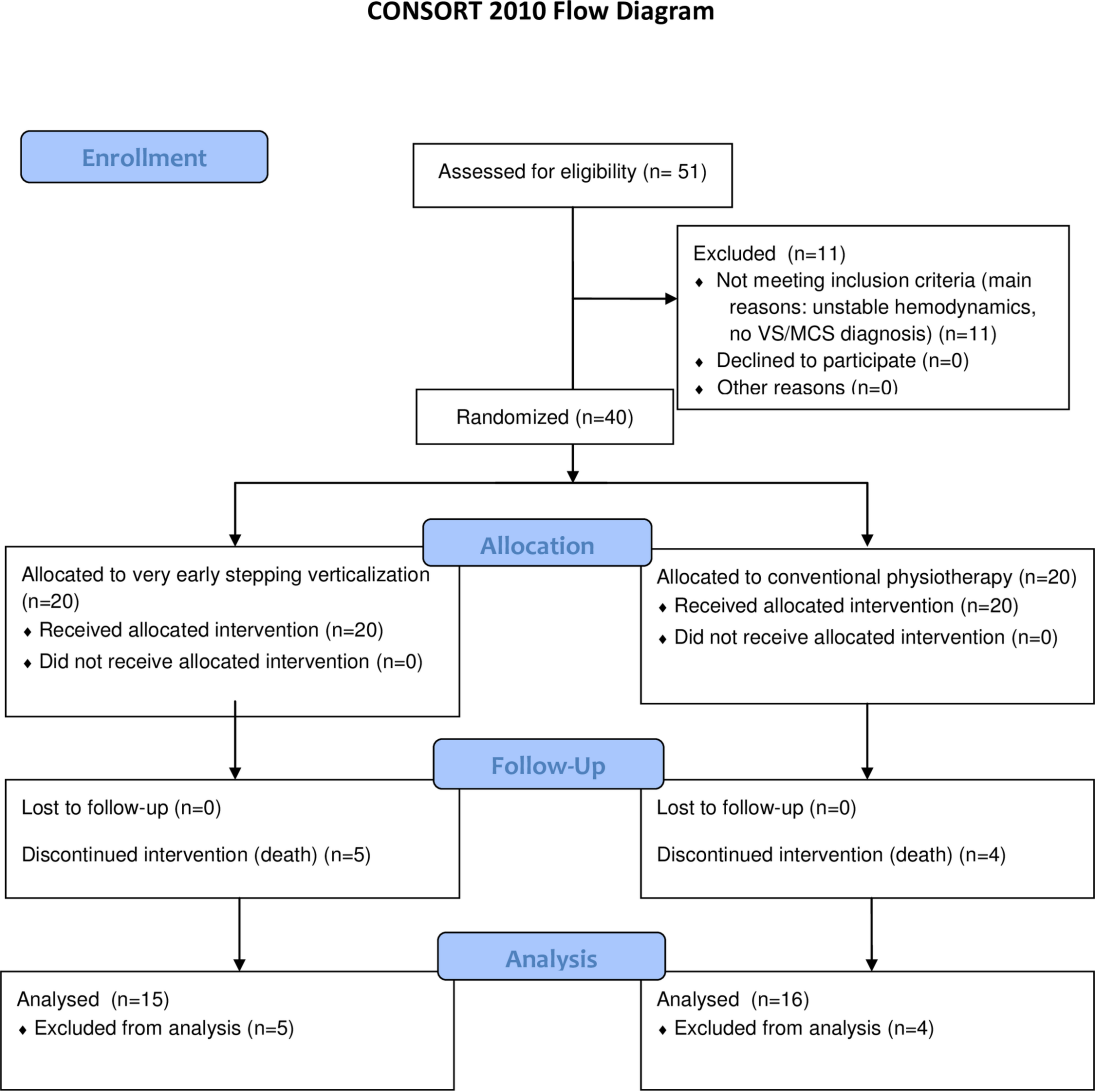
CONSORT flow diagram for the early stepping verticalization study. VS = Vegetative State; MCS: Minimally Conscious State

Fig 1 (CONSORT flow chart) shows the trial profile. A total of 40 patients were enrolled in the study. No adverse events during the stepping verticalization sessions nor changes in inclusion/exclusion criteria during the experimental treatment have occurred in any patient, so none of them has needed to interrupt any session or has required treatment interruption. No severe adverse event requiring withdrawal from the study have happened. Two patients in the early verticalization group and one in the control group died during their stay in ICU. Three more patients died in each group during the Neurorehabilitation period. Hence, the final study population analyzed consisted of 31 patients: 15 in the experimental and 16 in the control group respectively. All the 15 patients in the experimental group completed the 15 Erigo sessions.

Table 1 reports demographic and clinical data for the two groups of patients that completed the study. Baseline characteristics were not significantly different between groups, except for age and hypertension.

**Table 1:** Baseline characteristics of the population.

	Early verticalization (N = 15)	Controls (N = 16)	p-value
**Age (years)**	53±15	69±16	0.002
**Sex (M/F)**	9/6	11/5	0.72
**Etiology**			0.73
***Trauma***	7 (46.7)	5 (31.2)	
***Ischemia***	1 (6.7)	1 (6.2)	
***Hemorrhage***	5 (33.3)	9 (56.2)	
***Anoxia***	2 (13.3)	1 (6.2)	
**Main brain damage site**			0.61
***Diffuse***	6 (40)	3 (18.7)	
***Right hemisphere***	4 (26.7)	4 (25)	
***Left hemisphere***	2 (13.3)	6 (37.5)	
***Bilateral***	2 (13.3)	2 (12.5)	
***Cerebellar***	1 (6.7)	1 (6.2)	
**Risk factors**			
***Hypertension***	4 (26.7)	11 (68.7)	0.032
***Diabetes***	1 (6.7)	1 (6.2)	1.00
***Current smoking***	2 (13.3)	3 (18.7)	1.00
***Alcoholism***	2 (13.3)	3 (18.7)	1.00
***Obesity***	2 (13.3)	1 (6.2)	0.59

Data are given as mean±SD or N (%) for categorical variables.

Patients in the early verticalization group started stepping verticalization 12.4 ±7.3 days (between day 3 and day 30) after ABI. Due to the study protocol, these patients stayed in ICU long after the achievement of clinical stability. This led to a significantly different time spent in ICU (38.8 ± 15.7 vs 25.1 ± 11.2 days, p = 0.01) in the two groups. The following length of stay in the Neurorehabilitation unit was not statistically different among groups (114.4 ± 56.9 days for the experimental group vs 106.1 ± 57.3 days for the control group, p = 0.70). Seven patients in the experimental group and seven in the control group had a length of stay in Neurorehabilitation shorter than 100 days, due to good outcome (5 in both groups) or to patient transfer or family reasons (2 in both groups). The extra time in ICU was carried on to the total rehabilitation time (153.2 ± 59.6 days for patients in the early verticalization group vs 134.0 ± 61.0 days for controls, p = 0.41). However, the difference in the total rehabilitation time, besides being statistically not significant, was also not relevant from the clinical point of view, being about 14% of the total rehabilitation time.

Table 2 reports the outcome variables assessed at the three observation times. No statistically significant differences were observed in the two groups, but at ICU admission patients in the early stepping verticalization group tended to have a more compromised GCS (p = 0.024, borderline significant after Benjamini-Hochberg adjustment). At rehabilitation discharge 11 patients in the experimental group (73.3%) and 7 in the control group (43.75%) reached the maximum CRSr score (23/23).

**Table 2:** Outcome variables at the three observation times for the two groups.

	Early verticalization (N = 15)	Controls (N = 16)	p-value
**GCS**			
**T0**	7.0 (4.1,8.0)	8.5 (6.3,10.0)	0.024
**T1**	12.0 (8.1,14.8)	10.5 (7.7,14.0)	0.576
**T2**	15.0 (10.4,15.0)	13.0 (11.3,15.0)	0.348
**DRS**			
**T0**	25.0 (22.0,28.0)	23.0 (17.7,27.5)	0.189
**T1**	12.0 (10.0,24.0)	22.0 (11.0,27.0)	0.411
**T2**	6.0 (2.1,19.5)	15.5 (4.3,25.0)	0.234
**CRSr**			
**T0**	4.0 (3.0,5.7)	5.0 (3.0,12.0)	0.300
**T1**	19.0 (5.0,20.8)	10.5 (3.3,18.0)	0.099
**T2**	23.0 (9.4,23.0)	13.0 (7.0,23.0)	0.279
**LCF**			
**T0**	2.0 (1.0,3.0)	2.0 (1.0,3.0)	0.564
**T1**	4.0 (2.0,4.0)	3.0 (1.3,4.0)	0.432
**T2**	7.0 (3.1,7.0)	3.5 (3.0,7.0)	0.391

T0: admission. T1: ICU discharge. T2: Neurorehabilitation discharge. Data are given as median (lower, upper quartile). The p-values reported are pertaining to between groups comparisons at each observation time. GCS score ranges from 3 (worst) to 15 (best). DRS score ranges from 0 (best) to 29 (worst). CRSr score ranges from 0 (worst) to 23 (best). LCF score ranges from 1 (worst) to 7 (best).

In Table 3 the differences between measurements at rehabilitation discharge and ICU admission (T2-T0), between measurements at the ICU discharge and admission (T1-T0) and between measurements at rehabilitation discharge and ICU discharge (T2-T1) are given.

**Table 3:** Differences (Δ) between outcome variables measurements at the three observation times.

	Early verticalization (N = 15)	Controls (N = 16)	p-value
**GCS**			
**Δ (T2 –T0)**	7.0 (3.2,10.0)	4.5 (3.0,6.5)	0.076
**Δ (T1 –T0)**	4.0 (1.0,8.0)	2.5 (1.0,3.5)	0.068
**Δ (T2 –T1)**	1.0 (0.0,2.7)	2.5 (0.3,4.0)	0.365
**DRS**			
**Δ (T2 –T0)**	-20.0 (-22.0,-4.5)	-6.0 (-12.7,-2.0)	0.040
**Δ (T1 –T0)**	-5.0 (-16.0,-1.3)	-1.5 (-4.7,-0.5)	0.058
**Δ (T2 –T1)**	-4.0 (-9.0,-2.3)	-3.5 (-8.6,-1.0)	0.310
**CRSr**			
**Δ (T2 –T0)**	17.0 (5.1,18.8)	5.0 (2.3,11.0)	0.033
**Δ (T1 –T0)**	12.0 (2.0,15.8)	1.5 (0.3,4.0)	0.006
**Δ (T2 –T1)**	3.0 (0.1,4.0)	3.5 (1.0,5.5)	0.511
**LCF**			
**Δ (T2 –T0)**	4.0 (1.0,5.0)	2.5 (1.0,4.0)	0.135
**Δ (T1 –T0)**	1.0 (0.0,2.7)	1.0 (0.0,1.0)	0.265
**Δ (T2 –T1)**	2.0 (1.0,3.7)	2.0 (0.3,3.0)	0.418

**Δ** (T2-T0): values at rehabilitation discharge minus values at ICU admission. **Δ** (T1-T0): values at ICU discharge minus values at ICU admission. **Δ** (T2-T1): values at Neurorehabilitation discharge minus values at ICU discharge. Data are given as median (lower, upper quartile). The p-values reported are pertaining to between groups comparisons of the change (**Δ**) in outcome parameter for each couple of observation time. GCS score ranges from 3 (worst) to 15 (best). DRS score ranges from 0 (best) to 29 (worst). CRSr score ranges from 0 (worst) to 23 (best). LCF score ranges from 1 (worst) to 7 (best).

All outcome measures significantly improved in both groups after the overall treatment period (T2 vs T0, p<0.001 all). Comparing the improvement between groups (pertaining p values are reported in the last column of Table 3), a trend toward a better improvement in the early stepping verticalization group was observed in all outcome variables, but the difference was significant after Benjamini-Hochberg adjustment only for CRSr (p = 0.033) and borderline significant for DRS (p = 0.040).

Considering only the time spent in ICU, all outcome measures significantly improved in both groups after this period (T1 vs T0, p<0.004 all). This improvement was significantly better after Benjamini-Hochberg adjustment in the early stepping verticalization group only for CRSr (p = 0.006), while no significant differences in improvement were observed for GCS, DRS and LCF.

Similarly, all outcome measures significantly improved in both groups after the time spent in Neurorehabilitation (T2 vs T1, p<0.004 all) but no statistically significant differences in the improvement was found between groups in any outcome variable.

## Discussion

**Fig 2:**
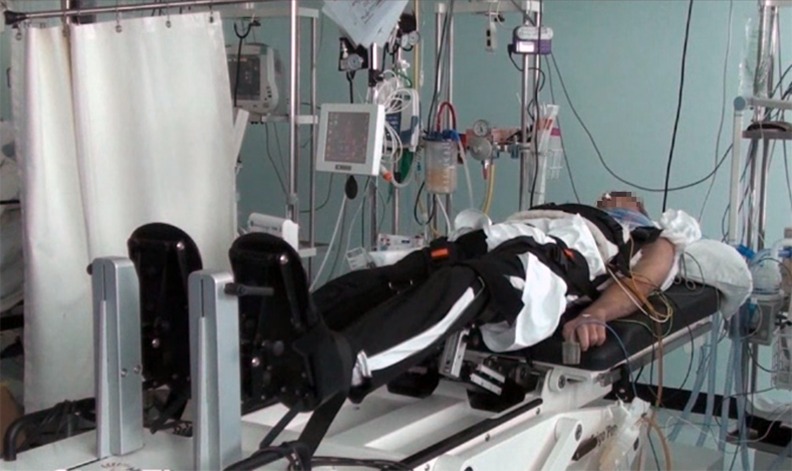
“Erigo” setting in the ICU room.

**Fig 3:**
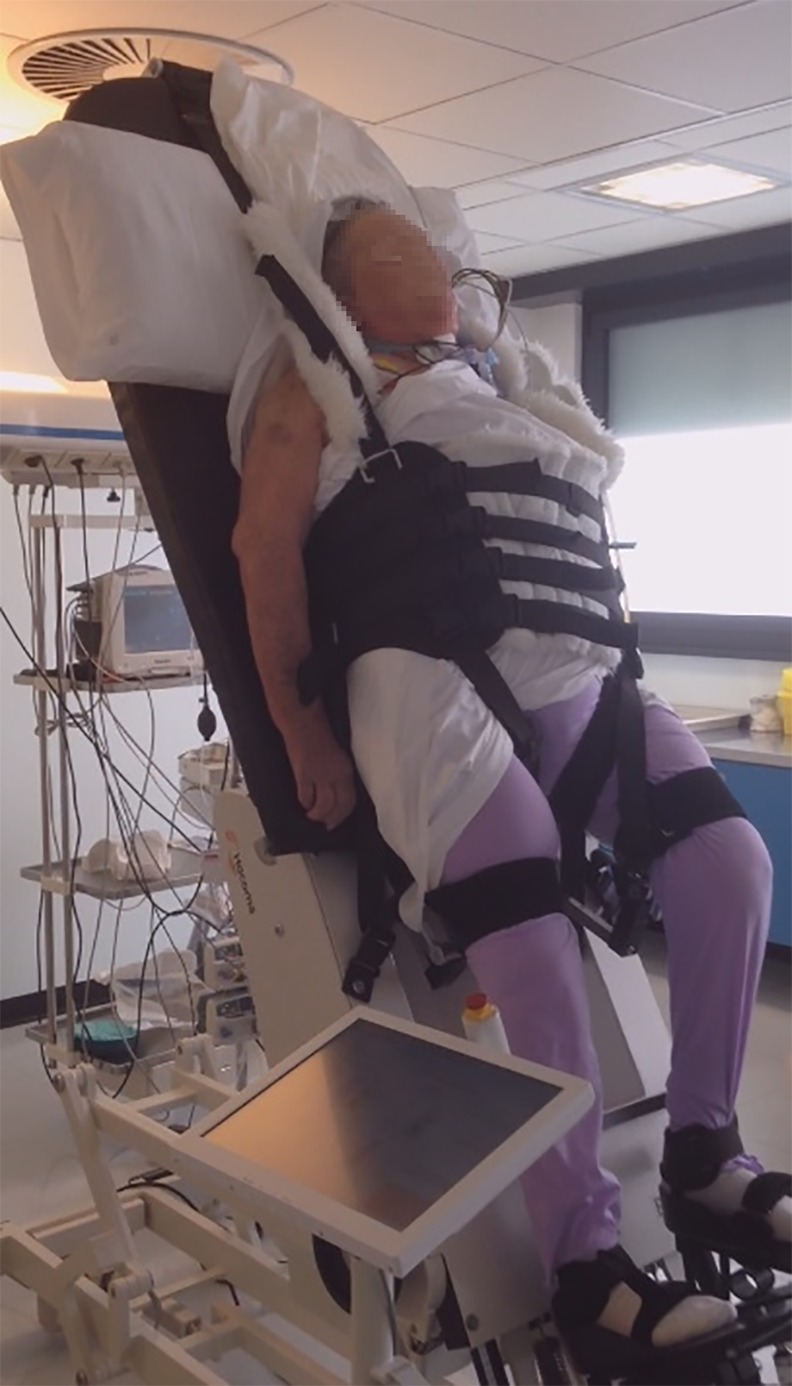
Stepping verticalization treatment in ICU.

To the best of our knowledge, this is the first study that evaluates the effects of a very early verticalization with leg stepping movements program on the functional and neurological outcome of patients with severe DOC. Four studies in the literature already showed that patients’ verticalization with a tilt table is able to induce an improvement on the level of consciousness. Elliott et al. assessed, using the Wessex Head Injury Matrix, the behavior of 12 VS and MCS patients while lying in bed and during a 20-minute period of standing with a standard tilt table. They attested consistent improvements in the highest ranked behavior and total number of behaviors in the standing position [20]. Riberholt et al. studied the effects of verticalization with a normal tilt table in 16 patients with VS/MCS within the first 3 months from injury. Despite the majority of patients needed to interrupt the sessions because of the occurrence of orthostatic intolerance, the authors observed an increased level of arousal (time with eyes open) in the upright position when compared to the supine one [21]. In comparison to the two abovementioned reports, that evaluate only the effect of single sessions of verticalization, our present study helps to give clinical implication to the previous findings, integrating the procedure into the rehabilitative treatment of DOC patients in ICU (Figs 2 and 3). With the same purpose, Toccolini et al. treated 23 mechanically ventilated patients with daily sessions of gradual verticalization with a standard tilt table in ICU. The patients showed a significant improvement of GCS and Richmond Agitation Sedation Scale scores during tilting between the beginning and the end of treatment [22]. Moreover, Krewer et al. compared the effects of 10 sessions of verticalization over 3 weeks with a standard tilt table or a tilt table with an integrated stepping device on CRSr scores of VS/MCS patients. They enrolled 50 patients between 1 to 6 months from injury and assessed the CRSr scores at baseline, after the 3 weeks of treatment and after 3 weeks of follow up and showed a better recovery with the standard tilt table [23]. The essential difference of our protocol lies in the verticalization timing (within the first month from the injury), in the use of a tilt table with integrated robotic stepping device in the rehabilitative treatment in comparison to standard physiotherapy alone and in the follow up timing (average of 4 months after the completion of the protocol).

Our patients were selected if diagnosed as VS or MCS on the third day after the ABI to be sure of the presence of a severe DOC. The similarity of the two groups according to sex, etiology, site of the main brain damage, comorbidities, LCF, DRS and CRSr scores at admission and number of deaths during the hospitalization makes the samples homogeneous. However, we found that the control group was significantly older (as a consequence, it was more affected by hypertension) while the experimental group had a lower GCS score at admission. Both older age and lower GCS scores at admission were previously reported to be independent predictors of a less favorable outcome in TBI patients [28–31].

According to the well-known adverse events [21,24–25] and considering the acute phase of the brain injury, we decided not to create a group of patients treated with a standard tilt-table. Our safety and feasibility study has already shown that the stepping verticalization of unconscious patients after TBI, even before the complete stabilization of the medical and neurological clinical state, is safe and feasible in ICU [26]. The present study confirms the same findings, but it broadens its research to all types of ABIs: patients started the treatment after 12.4 ± 7.3 days from the event, and none experienced orthostatic intolerance. Orthostatic hypotension was probably limited by: i) the precocity of the intervention, thus the shortness of the head-down bed rest, not enough to alter the autonomic and endocrine functions [22,26], ii) the rhythmic stepping movement of the lower limbs, able to lower the cardiovascular parameters fluctuation and the consequent risk of syncope in standing position [24,26].

Our results point out that at the end of the ICU stay (short-term outcome) both groups showed a significant improvement in all the tested scales (GCS, DRS, CRSr, LCF). However, when compared to the standard physiotherapy care, the early stepping verticalization was able to improve the CRSr score in a greater way. The DRS improvement was also higher in the experimental group, but without reaching statistical significance. On the other hand, GCS and LCF changes were not different, probably because of their lower sensitivity in the neurological assessment.

The following hospitalization in Neurorehabilitation was effective in giving an additional improvement of all the parameters in both groups, without any significant difference between groups. In this period, all patients received the individualized best rehabilitative treatment that our department can offer, without any differentiation in groups. At discharge from the Neurorehabilitation we observed in the experimental group a greater number of patients reaching the CRS-r maximum score. This “high responders”, compared to patients with lower CRSr scores at T2, showed in both groups a younger age, a lower incidence of hypertension and higher CRSr scores at T0 (data not shown).The global improvement (between the first evaluation in ICU and the end of the Neurorehabilitation stay) of the scores was significant for both groups. Nevertheless, we observed better long-term results in the experimental group, with significant higher improvement of CRSr and DRS and non-significant higher improvement of GCS and LCF. Therefore we can affirm that, considering the absence of a difference between groups in the improvement obtained in the Neurorehabillitation phase, the initial gain obtained by the experimental group in the ICU phase from the stepping-verticalization treatment was kept until the discharge from the Neurorehabilitation Unit (an average of 4 months follow up).

Patients in the experimental group showed a significantly longer length of stay in ICU (13 days on average). This is due to their necessity to complete the stepping verticalization protocol before being moved to the Neurorehabilitation Unit. Considering the duration of the protocol (3 weeks) and its beginning between the 3rd and the 30th day from the event, it happened that patients of the experimental group stayed in ICU long after the achievement of their clinical stability (main criterion for ICU discharge in both groups). Even though this might have partly contributed to the better short-term outcome in the experimental group, the overall rehabilitation time did not significantly differ, and should not have influenced the long-term outcome.

The better improvement in the experimental group may be explained by sensorial stimulation due to orthostatic position: orthostatism could indeed activate the proprioceptive, tactile, and vestibular pathways in comatose patients, leading to an increased cortical activation [32]. Another mechanism contributing to the neurological improvement after head-up tilt could be the lowering of ICP. Postural changes are known to alter the ICP, by redistributing the cerebrospinal fluid (CSF) within the craniospinal space [33] and modifying the venous outflow (thus the cerebral blood volume) through the valveless jugular veins [34–35]. However, head elevation in trauma patients was reported to be dangerous because of a marked decrease of mean arterial pressure (MAP), if not balanced by the activation of the cerebral autoregulation mechanisms, with consequent reduction in CPP [36]. On the other hand, a study performed with transcranial Doppler on patients with cerebral vasospasm after subarachnoid hemorrhage did not show any significant change in cerebral blood flow (CBF) after gradual head of bed elevation to 45° [37].

In conclusion, our positive findings on the long-term outcome support the use of a tilt table with robotic stepping device in the context of an early rehabilitative program of DOC patients.

## Study Limitations

This study has some limitations worthy of being addressed.

A study about the efficacy of a very early treatment on patients in the acute phase of an ABI, whose clinical evolution is highly variable before reaching a stability of the DOC, is always limited by the possible occurrence of an unverifiable spontaneous improvement. However, in our case, the use of a randomized trial reduced the power of this bias and increased the reliability of the results.

The same clinical variability, together with the study design, limited the homogeneity of the length of stay in ICU and in Neurorehabilitation unit between the two groups. Nevertheless, as discussed before, this did not affect the total time frame nor the consequent significance of the long term results.

The sample size was rather small, suggesting that our study might be better considered as a pilot trial. Indeed, the trends observed in the outcome variables, which in some cases could not be substantiated by statistical significance, foster the planning of large multicenter studies.Another limit was the absence of an ICP, CPP or CBF assessment during the sessions. However, exclusion criteria for our protocol were the presence of unstable ICP and a low CPP before starting the treatment. Considering that our patient did not develop secondary brain damages (e.g. new ischemic lesions) and significant MAP changes during and after the treatment, we can reasonably exclude a deterioration of the cerebral perfusion. Future studies about the trend of the abovementioned parameters during stepping verticalization would then be useful to clarify the mechanism of neurological improvement in DOC patients.

## Conclusion

The present study shows as an intensive stepping verticalization protocol, started since the acute stages of a severe ABI, improves the short-term and, more convincingly, long-term functional and neurological outcome of patients with DOC. We therefore propose the use of a tilt table with robotic stepping device for the rehabilitation of this group of patients since the first days of hospitalization in ICU, upon the reaching of hemodynamic, respiratory and intracranial stability.

## Supporting Information

S1 CONSORT Checklist CONSORT checklist. (DOCX)

S1 File Patients data for statistical analysis. (XLS)

S1 Protocol Original Protocol, Italian language. (PDF)

S2 Protocol English summary. (DOCX)

S1 Video Stepping verticalization procedure in ICU. The video shows a 30-minute session of stepping verticalization of a patient in vegetative state. The session is carried out in an ICU room, using a tilt table with integrated robotic stepping device. Cardiovascular and respiratory parameters are continuously monitored. Ventilator dependence, tracheal cannula, nasogastric tube and central venous catheter are not barrier to the treatment. See text for details.
(MPG)
