# Supplementary material for: Effectiveness of a Very Early Stepping Verticalization Protocol in Severe Acquired Brain Injured Patients: A Randomized Pilot Study in ICU
Source: PLoS One. 2016 Jul 22;11(7):e0158030. doi: 10.1371/journal.pone.0158030 (PMC4957764; doi:10.1371/journal.pone.0158030)
Supplement: S1 CONSORT Checklist — (DOCX) [file pone.0158030.s001.docx]

| **Section/topic** | **Item number** | **Checklist item** | **Reported on page number** |
| --- | --- | --- | --- |
| **Title and abstract** | 1a | Identification as a randomised trial in the title | **1** |
|  | 1b | Structured summary of trial design, methods, results, and conclusions | **2** |
| **Introduction** |  |  |  |
| Background and objectives | 2a | Scientific background and explanation of rationale | **3-4** |
|  | 2b | Specific objectives or hypotheses | **4** |
| **Methods** |  |  |  |
| Trial design | 3a | Description of trial design (such as parallel, factorial) including allocation ratio | **5-6** |
|  | 3b | Important changes to methods after trial commencement (such as eligibility criteria), with reasons | **-** |
| Participants | 4a | Eligibility criteria for participants | **5-6** |
|  | 4b | Settings and locations where the data were collected | **5** |
| Interventions | 5 | The interventions for each group with sufficient details to allow replication, including how and when they were actually administered | **6-8** |
| Outcomes | 6a | Completely defined prespecified primary and secondary outcome measures, including how and when they were assessed | **8** |
|  | 6b | Any changes to trial outcomes after the trial commenced, with reasons | **-** |
| Sample size | 7a | How sample size was determined | **8-9** |
|  | 7b | When applicable, explanation of any interim analyses and stopping guidelines | **-** |
| **Randomisation** |  |  |  |
| Sequence generation | 8a | Method used to generate the random allocation sequence | **6** |
|  | 8b | Type of randomisation; details of any restriction (such as blocking and block size) | **6** |
| Allocation concealment mechanism | 9 | Mechanism used to implement the random allocation sequence (such as sequentially numbered containers), describing any steps taken to conceal the sequence until interventions were assigned | **-** |
| Implementation | 10 | Who generated the random allocation sequence, who enrolled participants, and who assigned participants to interventions | **5-6** |
| Blinding | 11a | If done, who was blinded after assignment to interventions (for example, participants, care providers, those assessing outcomes) and how | **6** |
|  | 11b | If relevant, description of the similarity of interventions | **-** |
| Statistical methods | 12a | Statistical methods used to compare groups for primary and secondary outcomes | **9** |
|  | 12b | Methods for additional analyses, such as subgroup analyses and adjusted analyses | **9** |
| **Results** |  |  |  |
| Participant flow | 13a | For each group, the numbers of participants who were randomly assigned, received intended treatment, and were analysed for the primary outcome | **10** |
|  | 13b | For each group, losses and exclusions after randomisation, together with reasons | **10** |
| Recruitment | 14a | Dates defining the periods of recruitment and follow-up | **5 and 8** |
|  | 14b | Why the trial ended or was stopped | **-** |
| Baseline data | 15 | A table showing baseline demographic and clinical characteristics for each group | **12** |
| Numbers analysed | 16 | For each group, number of participants (denominator) included in each analysis and whether the analysis was by original assigned groups | **10** |
| Outcomes and estimation | 17a | For each primary and secondary outcome, results for each group, and the estimated effect size and its precision (such as 95% CI) | **11** |
|  | 17b | For binary outcomes, presentation of both absolute and relative effect sizes is recommended | **-** |
| Ancillary analyses | 18 | Results of any other analyses performed, including subgroup analyses and adjusted analyses, distinguishing prespecified from exploratory | **10-11** |
| Harms | 19 | All important harms or unintended effects in each group | **10** |
| **Discussion** |  |  |  |
| Limitations | 20 | Trial limitations, addressing sources of potential bias, imprecision, and, if relevant, multiplicity of analyses | **19** |
| Generalisability | 21 | Generalisability (external validity, applicability) of the trial findings | **19** |
| Interpretation | 22 | Interpretation consistent with results, balancing benefits and harms, and considering other relevant evidence | **16-18** |
| **Other information** |  |  |  |
| Registration | 23 | Registration number and name of trial registry | **5** |
| Protocol | 24 | Where the full trial protocol can be accessed, if available | **5** |
| Funding | 25 | Sources of funding and other support (such as supply of drugs), role of funders | **online** |
