# Supplementary material for: Effectiveness of a Very Early Stepping Verticalization Protocol in Severe Acquired Brain Injured Patients: A Randomized Pilot Study in ICU
Source: PLoS One. 2016 Jul 22;11(7):e0158030. doi: 10.1371/journal.pone.0158030 (PMC4957764; doi:10.1371/journal.pone.0158030)
Supplement: S1 Protocol — (PDF) [file pone.0158030.s003.pdf]

Regione Lombardia

ASL Como

COMITATO ETICO

Cl.: VII/03/03 – Fasc. VII/03 - 6
Trasmissione a mezzo telefax n. 0344/85896

ASL COMO
PROTOCOLLO GENERALE
N. 0066648 del 23/10/2012
Class: VII/03/03
0003773147

Al Direttore Generale
dell'Ospedale Moriggia Pelascini
dott.ssa Carla Nanni

22015 Gravedona ed Uniti CO

Oggetto: studio pilota di sicurezza e fattibilità: "La verticalizzazione precoce, attiva e passiva, mediante l'utilizzo dell'apparecchiatura ERIGO HOCOMA, nei pazienti affetti da CGLA in Neurorianimazione. Trasmissione parere.

In relazione allo studio in oggetto si trasmette, accluso alla presente, il parere reso dal Comitato Etico nella seduta dell'11.10.2012.

Cordiali saluti.

IL PRESIDENTE
dr. Domenico Santoro

ASL COMO
PROTOCOLLO GENERALE
N. 0066648 del 23/10/2012
Class: VII/03/03
000377314 7

COMITATO ETICO

PARERE N. 06/2012

Il giorno 11 ottobre 2012, alle ore 14,30 presso il presidio dell'ASL della Provincia di Como di via Castelnuovo, si è riunito il Comitato Etico dell'Azienda Sanitaria Locale della Provincia di Como.

La seduta è stata regolarmente indetta con comunicazione del 9.10.2012 prot. 0063540.

Sono presenti:

- dr. Domenico Santoro, (Presidente), Responsabile della U.O. Malattie Infettive dell'Azienda Ospedaliera Sant'Anna di Como;
- Sig. Gianstefano Buzzi, Rappresentante Associaz. Volontariato;
- dr. Gabriele Giussani – Pediatra di Famiglia;
- dr.ssa Amneris Magella, Responsabile del Servizio Medicina Legale
- dr.ssa Elena Caterina Monti, farmacologa (Università Insubria di Varese);
- dr Maurizio Ravera, Medico di Medicina Generale;
- dr. Paolo Rossitto, Medico specialista in anestesia e rianimazione;
- dr. Corrado Zuliani, Responsabile Servizio Assistenza Farmaceutica.

Per la Segreteria del Comitato Etico sono presenti:

- dr.ssa Piera Manfreda, Responsabile area coord. Affari Istituzionali;

Titolo dello studio: La verticalizzazione precoce, attiva e passiva, mediante l'utilizzo dell'apparecchiatura ERIGO HOCOMA, nei pazienti affetti da GCLA (grave cerebro lesione acquisita) in Neurorianimazione: studio pilota di sicurezza e fattibilità.

Natura dello studio: studio di fattibilità e sicurezza con dispositivo ERIGO HOCOMA.

Promotore dello studio: Ospedale Moriggia Pelascini di Gravedona.

Centro coinvolto nel progetto: Ospedale Moriggia Pelascini – UO Rianimazione/Terapia Intensiva.

IL COMITATO ETICO

DELL'ASL DELLA PROVINCIA DI COMO

Sentito il Componente del Comitato Etico, relatore designato per l'esposizione del Protocollo di Studio, esaminata la seguente documentazione prodotta dal promotore:

- richiesta di parere prot. 0046307 del 17.7.2012;
  1. presentazione dell'apparecchiatura Erigo Hocoma;
  2. scheda tecnica dispositivo;
  3. scale di valutazione utilizzate per l'inquadramento del paziente (Coma Recovery Scale Revised; Glasgow Coma Scale; Scala di Asworth modificata);
  4. consenso informato;
  5. scheda raccolta dati;
  6. scheda informativa paziente;
  7. protocollo di studio 24 aprile 2012
  8. Sinossi dello studio;

Dato atto che il Comitato Etico nella seduta del 13.9.2012 ha sospeso i termini per il rilascio del parere, avendo formulato con nota prot. 0060044 del 25.9.2012 alcune osservazioni in merito al Consenso Informato e alla Polizza Assicurativa.

Dato altresì atto che nella medesima del 13 settembre è stato sentito il Responsabile della UO di Rianimazione/Terapia Intensiva dr. Roberto Valsecchi, principal investigator, che ha illustrato i contenuti e le finalità dello studio.

Visti i curricula vitae del dr. Valsecchi e dei co-investigators indicati nel protocollo di studio.

Vista la seguente documentazione tecnico-scientifica pervenuta il 10.10.2012:
- protocollo di studio aggiornamento Ottobre 2012
- consenso informato per i pazienti incapaci di dare validamente il consenso informato;
- copia Polizza generale n. 2012RCG00008-562069;
- copia Appendice n. 2 alla polizza n. 2012RCG00008-562069

ad unanimità esprime

PARERE FAVOREVOLE

alla conduzione dello studio in oggetto presso la UO di Rinanimazione/Terapia intensiva dell'Ospedale Moriggia Pelascini di Gravedona, sotto la responsabilità del dr. Roberto Valsecchi, principal investigator, fermo restando il rispetto delle seguenti prescrizioni:

- come richiesto dalla normativa in materia di protezione dei dati personali, l'interessato dovrà dare il proprio consenso anche per il trattamento dei propri dati personali e sensibili, ai sensi del D.Lgs n. 196/2003 "Codice in materia di protezione dei dati personali";
- ai sensi del Decreto 14 luglio 2009 "Requisiti minimi per le polizze assicurative a tutela dei soggetti partecipanti alle sperimentazioni cliniche dei medicinali":
    - poiché l'appendice fa riferimento alla polizza base, la cui scadenza è prevista per il prossimo 1.1.2013, il promotore è tenuto, entro tale data, a presentare al Comitato Etico il nuovo certificato di rinnovo;
    - l'appendice deve fare espresso riferimento ai termini per la manifestazione danni e per la presentazione di richieste di risarcimento che non possono essere inferiori rispettivamente a 24 e 36 mesi dalla data di conclusione della sperimentazione, come previsto dal citato decreto (art. 1, comma 3);
    - non deve essere prevista una franchigia opponibile a terzi danneggiati.
- Il Comitato dovrà essere informato dell'inizio e della conclusione dello studio e di ogni eventuale emendamento.

- Si richiama l'attenzione alla scrupolosa osservazione di ogni evento avverso che si manifesti nel corso della sperimentazione, che dovrà essere comunicato secondo le modalità prevista dalla normativa vigente.

Il presente documento si compone di 02 pagine.

IL PRESIDENTE DEL COMITATO ETICO
dell'ASL della Provincia di Como
dr. Domenico Santoro

Parere n. 6/2012 dell'11.10.2012

Domenico Santoro

OSPEDALE GENERALE DI ZONA "MORIGGIA – PELASCINI"
Classificato ai sensi Art. 1 Legge n. 132 del 12 Febbraio 1968 con D.R. n. 6880 del 12 Febbraio 1974
22015 **GRAVEDONA ED UNITI (CO)** - ☎ (0344) 92.111 - FAX (0344) 85.896
UFFICIO PRENOTAZIONI ☎ (0344) 92.401
ITALIA HOSPITAL S.p.A.
Capitale Sociale Euro 1.033.000,00 i.v. - Sede Legale: Corso Vittorio Emanuele II, 87 - 00186 ROMA
Ufficio Registro Imprese: Roma - RM - 1999 - 145653 - Codice Fiscale e Partita I.V.A. 05780911003

La verticalizzazione precoce, attiva e passiva, mediante l'utilizzo dell'apparecchiatura ERIGO HOCOMA, nei pazienti affetti da GCLA (grave cerebro lesione acquisita) in Neurorianimazione: studio pilota di sicurezza e fattibilità.

Responsabili:

**Dott. Roberto Valsecchi**	**Investigatore principale**
**Dott. Luca Sebastianelli**	**Coinvestigatore**
**Dott. Viviana Versace**	**Coinvestigatore**
**Prof. Leopold Saltuari**	**Coinvestigatore**
**Prof. Marcus Kofle**	**Coinvestigatore**
**Prof. Giorgio Sandrini**	**Coinvestigatore**

Data del protocollo: **24 Aprile 2012**
Aggiornamento **ottobre 2012**

Sistema Sanitario Regione Lombardia

INDICE

INTRODUZIONE

Razionale dello studio e ipotesi di ricerca

DISEGNO DELLO STUDIO

OBIETTIVI DELLO STUDIO

Obiettivo primario

Obiettivi secondari

ENDPOINT DELLO STUDIO

Endpoint primario

Endpoint secondari

CRITERI DI ELEGGIBILITA'

Criteri di inclusione

Criteri di esclusione

VALUTAZIONE DEL PAZIENTE

Pre-trattamento

In corso di trattamento

Fine trattamento

METODO

RESPONSABILITA' DI APPLICAZIONE DEL PROTOCOLLO DI STUDIO

CONSIDERAZIONI ETICHE

ALLEGATI

BIBLIOGRAFIA

INTRODUZIONE

Razionale dello studio e ipotesi di ricerca

I traumi, le emorragie intracerebrali gravi, l'anossia e altre forme di lesioni cerebrali locali e diffuse possono portare al coma, stato vegetativo o ad uno stato di coscienza minima.

Per "grave cerebrolesione acquisita" (GCLA) si intende un danno cerebrale, di origine traumatica o di altra natura, tale da determinare una condizione di coma, più o meno protratto, e menomazioni senso-motorie, cognitive o comportamentali, che comportano disabilità grave.

Nell'Unità Operativa di Neurorianimazione dell'Ospedale di Gravedona, nel triennio 2009 – 2011 sono stati ricoverati 203 pazienti affetti da GCLA, di cui 60 reclutabili per lo studio.

L'incidenza di stato vegetativo protratto per almeno sei mesi è stimato, a seconda delle casistiche riportate in Letteratura pari a 5-25 per milione di abitanti (UK 5, USA 17, Francia 25 per milione di popolazione). Lo stato vegetativo viene definito come una condizione clinica caratterizzata dalla completa inconsapevolezza del sé e dell'ambiente, ma è accompagnata da cicli sonno-veglia con completa o conservazione parziale delle funzioni dell'asse ipotalamico e del tronco encefalico.

Un individuo affetto da GCLA necessita di ricovero ospedaliero per trattamenti rianimatori o neurochirurgici di durata variabile da alcuni giorni ad alcune settimane (fase acuta). Dopo questa fase, sono in genere necessari interventi medico-riabilitativi di tipo intensivo, anch'essi da effettuare in regime di ricovero ospedaliero, che possono durare da alcune settimane ad alcuni mesi (fase post-acuta o riabilitativa). Nella maggior parte dei casi, dopo la fase di ospedalizzazione, permangono sequele che rendono necessari interventi di carattere sanitario e sociale a lungo termine, volti ad affrontare menomazioni, disabilità persistenti e difficoltà di reinserimento famigliare, sociale, scolastico e lavorativo (fase del reinserimento o degli esiti). Gli esiti disabilitanti delle gravi cerebrolesioni, in particolare di quelle traumatiche, costituiscono un problema di particolare rilevanza sanitaria e sociale nel nostro paese, come nella maggior parte delle nazioni industrializzate.

E' stato dimostrato che la riabilitazione motoria precoce effettuata quotidianamente può rallentare l'evoluzione verso la contrattura degli arti, inoltre la mobilitazione fuori dal letto e in particolare la mobilitazione in una posizione eretta può costituire sia una stimolazione che una cura preventiva. La verticalizzazione è stata spesso utilizzata nelle unità di terapia intensiva per aumentare la stimolazione cerebrale e ha dimostrato di migliorare la vigilanza e la coscienza in un piccolo gruppo di pazienti in stato vegetativo o in uno stato di coscienza minima.[i-ii]

La verticalizzazione precoce si è dimostrata efficace nel prevenire o rallentare lo sviluppo di complicazioni, come contratture ed anchilosi degli arti, osteoporosi o ulcere da decubito, ed efficace nel migliorare la ventilazione polmonare. [iii]

Anche se auspicabile, la verticalizzazione non può sempre essere facilmente praticata in pazienti che sono ancora incoscienti soprattutto nelle fasi precoci dopo un evento cerebrale acuto. Un problema comune è la presenza di sincopi a causa di una disfunzione del sistema simpatico e l'assenza del meccanismo di "pompa venosa" per la paralisi dei muscoli delle gambe.

In passato, quindi, la verticalizzazione è stata eseguita gradualmente in un periodo di diverse settimane, per stabilizzare il sistema circolatorio e ridurre le sincopi ricorrenti. Recentemente, tuttavia, è stato dimostrato, in pazienti con lesioni del midollo spinale, che le stimolazione elettriche e l'esercizio passivo delle gambe sono in grado di migliorare e rafforzare la risposta cardiovascolare nella verticalizzazione e di stabilizzare la circolazione. [iv-v]

E' stato inoltre dimostrato che il movimento di "stepping" in posizione eretta riduce il numero di sincopi.[4]

L'utilizzo di una tavola inclinata e di un sistema robotico integrato per lo "stepping" è uno strumento utile per indurre simultaneamente movimenti passivi delle gambe e contemporanea verticalizzazione in pazienti in coma o in stato semi-comatoso, dopo lesioni cerebrali.

L'Erigo (Hocoma Inc., Zurich, Switzerland) è un piano inclinabile a cui è stato integrato un sistema robotico di stepping, con il quale è possibile attuare un mobilizzazione passiva bilaterale degli arti inferiori in uno schema motorio che simula quello del cammino.[vi]

Il tronco del paziente è assicurato da un'imbragatura che lo fissa al piano da statica, mentre il movimento degli arti inferiori è assicurato da un motore a controllo computerizzato.

I piedi del paziente vengono fissati a due pedane mobili, che assecondano la flessione d'anca e il conseguente movimento in flessione di ginocchio. L'inclinazione della tavola può essere continuamente regolata da orizzontale fino alla posizione verticale. La velocità del movimento delle gambe è modificabile da 0 a 80 passi al minuto.

Dopo il superamento dell'evento acuto il sistema robotico Erigo Hocoma trova ulteriore applicazione, per esempio, nella riabilitazione post-stroke. Negli ultimi anni è stato introdotto il Treadmill; numerosi articoli [vii-viii] dimostrano la validità di questo "gait trainer", che consente la ripetizione del ciclo del passo effettuato sul posto, con o senza allevio di peso. Condizione necessaria per il conseguimento dei risultati è però l'assistenza di almeno un terapista, che manualmente deve controllare e correggere il movimento dell'arto paretico nelle varie fasi del passo. Nonostante l'assistenza del terapista, può comunque risultare molto complesso e faticoso garantire la simmetria del cammino.

Utilizzando un sistema robotico come Erigo Hocoma è invece possibile indurre un movimento degli arti inferiori del paziente nel modo più corretto.

Recenti pubblicazioni evidenziano come i sistemi automatizzati garantiscano una maggiore simmetria nel cammino [ix-x]e uno spostamento del centro di massa più fisiologico. A questo va aggiunta la miglior sensazione che il paziente riferisce rispetto all'assistenza manuale e la possibilità di effettuare numerosi steps (anche più di mille in una sessione di 30 min).

Studi Elettromiografici effettuati sul Treadmill, [xi-xii] ma anche su sistemi robotici di gait trainer [xiii] dimostrano l'esistenza di Centri Locomotori Spinali che generano appropriate attivazioni muscolari anche in assenza di un controllo encefalico. Queste attivazioni vengono generate da input afferenti derivanti dall'associazione della statica eretta e del parziale allevio di carico al movimento assistito degli arti inferiori. Inoltre è stato dimostrato come la plasticità dei Centri Locomotori Spinali sia direttamente correlata alla persistenza di input afferenti adeguati.[xiv]

Concetto fondamentale su cui si basa l'introduzione dell'Erigo nella riabilitazione in fase post acuta è l'influenza del movimento passivo sulla plasticità cerebrale .

Secondo studi effettuati da Weilner [xv]e successivamente da Nelles [xvi](utilizzando tecniche di Neuro-imaging quali la P.E.T. e la risonanza magnetica) la mobilizzazione passiva, prima della fase riabilitativa, produce gli stessi pattern di attivazione cerebrale del movimento attivo al termine del programma riabilitativo nei pazienti colpiti da post-stroke.[xvii-xviii]

DISEGNO DELLO STUDIO:

Saranno oggetto del nostro studio pazienti affetti da GCLA da *trauma cranico* e quelli con GCLA _da *stroke con grave compromissione dello stato di coscienza.*

Lo studio è di tipo prospettico senza gruppo di controllo e si configura come uno studio pilota.

Studieremo gli effetti della verticalizzazione precoce con sistema Erigo Hocoma in 5 pazienti affetti da GCLA ricoverati in Neurorianimazione.

Questo studio valuta la sicurezza e la fattibilità della verticalizzazione precoce con sistema robotizzato ERIGO HOCOMA.

Il presupposto clinico e teorico di questo studio è quello che tutti i pazienti affetti da GCLA, potrebbero giovare di un trattamento di verticalizzazione precoce attiva e passiva.

La verticalizzazione precoce in fase acuta di uno stroke con grave disturbo di coscienza o di un trauma cranico, in questo studio è intesa come la mobilizzazione del paziente non prima di tre (3) giorni e non oltre i trenta (30) giorni dall'evento indice.

In ogni caso la procedura di verticalizzazione in questi pazienti partirà da 20° per 3 min. Se non si verificheranno effetti indesiderati, si porterà il paziente a 40 gradi per 3 min. e quindi, se il quadro clinico rimane stabile, ad 60 gradi con un mantenimento della posizione per 20 minuti.

La frequenza di passo sarà costante di 20/minuto.

All'inizio del trattamento sarà effettuata una valutazione neurologica clinica e strumentale che si ripeterà al raggiungimento della massima verticalizzazione e a fine trattamento.

Sarà effettuata una rilevazione in continuo dei parametri monitorati .

Sarà motivo di sospensione della verticalizzazione se si verificherà anche una solo delle seguenti condizioni non altrimenti prontamente correggibili :

- ✓ Una variazione della frequenza cardiaca </= 40 min. o >/= 150 min.
- ✓ Una pressione arteriosa media (PAM) </= 70 mm/Hg
- ✓ Una SaO2 < 90%

La durata del trattamento sarà di 15 sedute con cadenza giornaliera.

La valutazione neurologica consisterà in un esame neurologico generale e nella somministrazione della Recovery Coma Scale – Revised e della scala di Ashworth.

Sarà inoltre valutata la risposta neurovegetativa mediante Sympathetic Skin Response.

La risoluzione della sedazione sarà sempre documentata con EEG.

I pazienti con *trauma cranico* saranno distinti in due gruppi:

- Pazienti con trauma cranico puro
- Pazienti con trauma cranico associato a politraumatismo

I pazienti con politraumatismo associato, saranno esclusi dallo studio. La presenza di eventuali piccole fratture segmentali, che comunque non rientrano nella definizione di politrauma, saranno valutate di volta in volta se condizionanti o meno la mobilizzazione ai fini dell'arruolamento.

Sia nei pazienti con trauma cranico che in quelli con stroke associato a grave compromissione dello stato di coscienza, una evenienza con frequenza non trascurabile, è rappresentata da un quadro di iniziale *ipertensione endocranica.*

Questo stato, sempre potenzialmente pericoloso per la sopravvivenza del paziente, sarà trattato secondo le Linee Guida Internazionali. 19, 20

Per l'arruolamento di questi pazienti, sarà previsto un attento monitoraggio della pressione intracranica che dovrà dimostrare una sua stabilizzazione senza il supporto di farmaci sedativi, ed al momento dell'arruolamento avere un valore </= 25 mm/Hg.

Nello studio sarà previsto per questi pazienti, il monitoraggio della ICP oltre che un monitoraggio cardiovascolare costante così da ottimizzare, in ogni istante della mobilizzazione, la CPP che dovrà mantenersi su valori >/= 70 mm/Hg.

Saranno esclusi dallo studio i pazienti in cui la pressione intracranica non sia stata ricondotta a valori compatibili con un range di normalità.

In tutti i pazienti saranno monitorati in continuo i seguenti parametri cardiocircolatori: elettrocardiogramma (ECG), frequenza cardiaca (HR), pressione arteriosa media (PAM), pressione venosa centrale (CVP), la gittata cardiaca (CO), l'indice cardiaco (CI), il volume di acqua polmonare extravascolare indicizzato (ELWI), il volume di sangue intratoracico indicizzato (ITBI), frazione di eiezione globale (GEF), le resistenze vascolari sistemiche indicizzate (SVRI), stroke volume indicizzato (SVI), variazioni di stroke volume (SVV), emogasanalisi arteriosa/venosa (EGA/V), la saturazione in ossigeno del sangue venoso misto centrale ($ScvO_2$), la SaO_2, la CO_2 di fine espirazione ($EtCO_2$).

Nei pazienti che hanno mostrato nella prima fase ipertensione endocranica, sarà mantenuto il monitoraggio della pressione intracranica (ICP) e della pressione di perfusione cerebrale (CPP).

Sistema Sanitario Regione Lombardia

OBIETTIVO DELLO STUDIO

Obbiettivo primario:

- L'obiettivo del presente studio è verificare la fattibilità della procedura in assoluta sicurezza per il paziente.

Obiettivi secondari:

- Valutare gli effetti della verticalizzazione precoce, attiva e passiva, con sistema Erigo Hocoma, nei due gruppi di pazienti affetti da GCLA secondaria a *traumi cranici* e accidenti di natura *vascolare con grave compromissione dello stato di coscienza.*

  La valutazione di tali effetti sarà effettuata su:

  - ✓ Parametri clinici: valutazione neurologica mediante Coma Recovery Scale e scala di Ashworth.
  - ✓ Parametri strumentali: tutti gli acquisiti.
  - ✓ Studio propedeutico ad un'indagine successiva finalizzata alla conferma dei risultati attesi

ENDPOINTS DELLO STUDIO:

Endpoints primari:

- Dimostrare la massima verticalizzazione raggiungibile in condizioni di sicurezza.

Endpoint secondario:

- Misurare gli effetti della verticalizzazione sui parametri strumentali acquisiti.

PRINCIPALI CRITERI DI INCLUSIONE/ESCLUSIONE

Verranno arruolati nello studio pazienti degenti nel Reparto di Neurorianimazione dell'Ospedale Generale di zona "Moriggia Pelascini" di Gravedona ed Uniti (CO).

I principali criteri di inclusione consistono:

1. Pazienti affetti da grave cerebrolesione acquisita (GCLA).
2. Età maggiore di 18 anni ed inferiore a 75 anni.
3. GCS all'ingresso < / = 8
4. Scambi respiratori adeguati con PaO2/FiO2 > / = 250
5. Assenza di sedazione maggiore.

I criteri di esclusione comprendono:

1. Trauma toracico maggiore comportante instabilità della gabbia toracica per presenza di fratture costali multiple e/o pneumotorace
2. Presenza di fratture agli arti inferiori e al bacino, lesioni vascolari, lesioni cutanee con perdita di sostanza a livello addominale, pelvico o degli arti inferiori
3. Trombosi venosa profonda
4. Instabilità cardiocircolatoria nonostante il supporto di amine
5. Pressione intracranica (PIC) non ben controllata ed instabile con valori superiori a 25 mm/Hg, e Pressione di Perfusione Cerebrale (PPC) con valori inferiori a 60 mm/Hg
6. Insufficienza renale severa che richieda trattamento sostitutivo
7. Insufficienza epatica scompensata
8. Valore di ematocrito </= 30 %
9. Peso corporeo > 135 kg o altezza > 210 cm

VALUTAZIONE DEL PAZIENTE
MATERIALI E METODI

Saranno valutati, per essere inclusi nel protocollo di studio, tutti i pazienti che:

- accederanno al reparto di Neurorianimazione dell'Ospedale di Gravedona ed Uniti per un evento determinante una GCLA
- rispetteranno i criteri di inclusione ed esclusione
- accetteranno di aderire al protocollo sottoscrivendo il consenso informato; nel caso di paziente privo di coscienza si farà riferimento ad un tutore legale.

La procedura di verticalizzazione con Erigo Hocoma sarà applicata a partire dal terzo giorno dall'evento indice e non oltre il trentesimo.

<u>Arruolamento paziente</u>

1. Eleggibilità del paziente in base ai criteri di inclusione e di esclusione
2. Valutazione clinico strumentale del paziente avente per obbiettivo la conferma di una sufficiente stabilità emodinamica e neurologica.
3. Screening ematochimico comprendente emocromo, quadro coagulativo,funzionalità renale ed epatica.
4. Valutazione strumentale neurologica mediante EEG e Sympathetic Skin Response
5. Valutazione neurologica clinica da parte dello Specialista Neurologo utilizzando la Coma Recovery Scale – Revised.

I dati raccolti saranno registrati su apposita scheda "raccolta dati" (vedi allegato) .

<u>Valutazioni in corso di trattamento</u>

Durante il trattamento, tutti i pazienti sono sottoposti ad un monitoraggio completo in continuo, secondo le modalità precedentemente illustrate.

Ai tempi previsti dal protocollo, sarà effettuata valutazione clinica neurologica ed una registrazione dei seguenti parametri cardiocircolatori : PAM, FC, SaO_2 e, dove prevista, PIC e CPP .

I parametri valutati saranno registrati sulla scheda raccolta dati del paziente includendo orari e tempi della procedura ed eventuali eventi avversi che hanno reso necessaria l'interruzione della procedura di verticalizzazione.

Valutazione di Fine trattamento

A fine trattamento, a partire dalla sesta ora e fino alla dodicesima, è prevista una valutazione neurologica clinica e strumentale (Sympathetic Skin Response) ed una registrazione di tutti i parametri cardiocircolatori sottoposti a valutazione in continuo: elettrocardiogramma (ECG), frequenza cardiaca (HR), pressione arteriosa media (MAP), pressione venosa centrale (CVP), indice cardiaco (CI), volume di sangue intratoracico indicizzato (ITB), volume di acqua polmonare extravascolare indicizzato (ELWI), indice di funzionalità cardiaca (CFI), resistenze vascolari sistemiche indicizzate (SVRI), stroke volume indicizzato (SVI), variazioni di stroke volume (SVV), emogasanalisi (EGA), CO2 di fine espirazione (EtCO2) e dove previsti, pressione intracranica (ICP), pressione di perfusione cerebrale (CPP).

Tutti i parametri saranno registrati sulla scheda raccolta dati del paziente includendo orari e tempi della procedura ed eventuali eventi avversi che hanno reso necessaria l'interruzione precoce della procedura di verticalizzazione.

RESPONSABILITA' DI APPLICAZIONE DEL PROTOCOLLO DI STUDIO

La responsabilità dell'arruolamento dei pazienti è condivisa dallo Sperimentatore Principale e dai coinvestigatori.

L'applicazione corretta del protocollo sarà garantita dai coinvestigatori.

CONSIDERAZIONI ETICHE

Lo studio verrà condotto in accordo con la dichiarazione di Helsinki e verrà preceduto dall'approvazione scritta ottenuta dal Comitato Etico. Verrà, inoltre, richiesto un consenso informato scritto ad ogni paziente o al tutore legale al momento della visita di screening.

DOCUMENTI ALLEGATI:

1) Presentazione Erigo Hocoma
2) Scheda tecnica Erigo Hocoma
3) Scale di valutazione utilizzate per l'inquadramento del paziente
    3 a – Coma Recovery Scale Revised
    3 b – Glasgow Coma Scale
    3 c – Scala di Asworth modificata
4) Scheda consenso informato
5) Scheda raccolta dati
6) Scheda informativa paziente

BIBLIOGRAFIA

[i] Elliot L, Coleman M, Shiel A et al. Effect of posture on levels of arousal and awareness in vegetative and minimally conscious patients: a preliminary investigation. J Neurol Neurosurg Psychiatry 2005; 76: 298–99.

[ii] Chang AT, Boots RJ, Hodges PW et al. Standing with the assistance of a tilt table improves minute ventilation in chronic critically ill patients. Arch Phys Med Rehabil 2004; 85: 1972–76)

[iii] Chang AT, Boots R, Hodges PW, Paratz J. Standing with assistance of a tilt table in intensive care: a survey of Australian physiotherapy practice. Aust J Physiother 2004; 50: 51–54

[iv] Faghri PD. Yount JP, Pesce WJ, Seethrama S, Votto JJ. Circulatory hypokinesis and functional electric stimulation during standing in persons with spinal cord injury. Arch Phys Med Rehabil 2001; 82: 1587–95.

[v] Muraki S, Ehara Y, Yamasaki M. Cardiovascular responses at the onset of passive leg cycle exercise in paraplegics with spinal cord injury. Eur J Appl Physiol 2000; 81: 271–74

[vi] Luther MS, Krewer C, Müller F, Koenig E. Comparison of orthostatic reactions of patients still unconscious within the first three months of brain injury on a tilt table with and without integrated stepping. A prospective, randomized crossover pilot trial. Clin Rehabil 2008; 22; 1034

[vii] McCain KJ, Pollo FE, Baum BS, Coleman SC, Baker S, Smith PS. Locomotor treadmill training with partial body-weight support before overground gait in adults with acute stroke: a pilot study. Arch Phys Med Rehabil. 2008 Apr;89(4):684-91

[viii] Visintin M, Barbeau H, Korner-Bitensky N, Mayo NE. A new approach to retrain gait in stroke patients through body weight support and treadmill stimulation. Stroke. 1998 Jun;29(6):1122-8

[ix] Werner C, Von Frankenberg S, Treig T, Konrad M, Hesse S. Treadmill training with partial body weight support and an electromechanical gait trainer for restoration of gait in subacute stroke patients: a randomized crossover study. Stroke. 2002 Dec;33(12):2895-901.

[x] Uhlenbrock D, Sarkodie-Gyan T, Reiter F, Konrad M, Hesse S. [Development of a gait trainer with regulated servo-drive for rehabilitation of locomotor disabledpatients]. Biomed Tech (Berl). 1997 Jul-Aug;42(7-8):196-202. German.

[xi] Dietz V, Colombo G, Jensen L. Locomotor activity in spinal man. Lancet. 1994 Nov 5;344(8932):1260-3.

[xii] Dietz V, Colombo G, Jensen L, Baumgartner L. Locomotor capacity of spinal cord in paraplegic patients. Ann Neurol. 1995 May;37(5):574-82.

[xiii] Hidler JM, Wall AE. Alterations in muscle activation patterns during robotic-assisted walking. Clin Biomech (Bristol, Avon). 2005 Feb;20(2):184-93.

[xiv] Dietz V. Body weight supported gait training: from laboratory to clinical setting. Brain Res Bull. 2008 Jul 30;76(5):459-63. Epub 2008 Mar 25. Review.

[xv] Weiller C, Jüptner M, Fellows S, Rijntjes M, Leonhardt G, Kiebel S, Müller S, Diener HC, Thilmann AF. Brain representation of active and passive movements. Neuroimage. 1996 Oct;4(2):105-10.

[xvi] Nelles G, Jentzen W, Jueptner M, Müller S, Diener HC. Arm training induced brain plasticity in stroke studied with serial positron emission tomography. Neuroimage. 2001 Jun;13(6 Pt 1):1146-54

[xvii] Cao Y, D'Olhaberriague L, Vikingstad EM, Levine SR, Welch KM. Pilot study of functional MRI to assess cerebral activation of motor function after poststroke hemiparesis. Stroke. 1998 Jan;29(1):112-22.

[xviii] Chollet F, DiPiero V, Wise RJ, Brooks DJ, Dolan RJ, Frackowiak RS. The functional anatomy of motor recovery after stroke in humans: a study with positron emission tomography. Ann Neurol. 1991 Jan;29(1):63-71.

[ix] The Brain Trauma Foundation,The American Association of Neurological Surgeons,the joint Section on Neurotrauma and Critical Care: guidelines for the Management of Severe Head injury, 1995.

[xx] Gruppo di Studio sul trauma cranico della SINCh. Guidelines for minor head injury patients' management in adult age. J Neurosurg sci 1996; 40: 11 - 15

OSPEDALE GENERALE DI ZONA "MORIGGIA – PELASCINI"
Classificato ai sensi Art. 1 Legge n. 132 del 12 Febbraio 1968 con D.R. n. 6880 del 12 Febbraio 1974
22015 **GRAVEDONA ED UNITI (CO)** - ☎ (0344) 92.111 - FAX (0344) 85.896
UFFICIO PRENOTAZIONI ☎ (0344) 92.401
ITALIA HOSPITAL S.p.A.
Capitale Sociale Euro 1.033.000,00 i.v. - Sede Legale: Corso Vittorio Emanuele II, 87 - 00186 ROMA
Ufficio Registro Imprese: Roma - RM - 1999 - 145653 - Codice Fiscale e Partita I.V.A. 05780911003

DICHIARAZIONE DI CONSENSO (soggetti incapaci)

Titolo dello studio: La verticalizzazione precoce, attiva e passiva, mediante l'utilizzo dell'apparecchiatura ERIGO HOCOMA, nei pazienti affetti da GCLA (grave cerebro lesione acquisita) in Neurorianimazione: studio pilota di sicurezza e fattibilità.

L'informazione al paziente in virtù della propedeuticità di tale fase dovrà essere fornita in un momento precedente e formalmente distinto dal recepimento del consenso.

Io sottoscritto ________________________________ dichiaro di aver ricevuto dal dottor ________________________________ in data ________________ spiegazioni esaurienti in merito alla richiesta di partecipazione del mio tutelato allo studio sperimentale in oggetto, secondo quanto riportato nel foglio informativo qui allegato, copia del quale mi è stata consegnata in data ________________

Dichiaro di aver potuto discutere tali spiegazioni, di aver potuto porre tutte le domande che ho ritenuto necessarie e di aver ricevuto in merito risposte soddisfacenti.

Dichiaro che anche il mio tutelato ha ricevuto informazioni sulla sperimentazione, sui suoi rischi e benefici, commisurate alla sua capacità di comprensione.

Accetto dunque liberamente che il mio tutelato partecipi alla sperimentazione, avendo compreso i rischi ed i benefici che sono implicati in questa partecipazione.

Mi sarà consegnata una lettera per il Medico di famiglia del mio tutelato che avrò cura di consegnargli qualora desideri informarlo.

Acconsento (o non acconsento) che il dottor …comunichi al mio medico di medicina generale dottor… quanto a me spiegato sul significato della ricerca cui prenderà parte il mio tutelato.

Sono stato inoltre informato del mio diritto ad avere libero accesso alla documentazione relativa alla sperimentazione ed alla valutazione espressa dal Comitato Etico.

Data ________________________________

Firma dell'esercente la tutela ________________________________

Data ________________________________

Firma del medico che ha informato l'esercente la tutela ________________________________

OSPEDALE GENERALE DI ZONA "MORIGGIA – PELASCINI"
Classificato ai sensi Art. 1 Legge n. 132 del 12 Febbraio 1968 con D.R. n. 6880 del 12 Febbraio 1974
22015 GRAVEDONA ED UNITI (CO) - ☎ (0344) 92.111 - FAX (0344) 85.896
UFFICIO PRENOTAZIONI ☎ (0344) 92.401
ITALIA HOSPITAL S.p.A.
Capitale Sociale Euro 1.033.000,00 i.v. - Sede Legale: Corso Vittorio Emanuele II, 87 - 00186 ROMA
Ufficio Registro Imprese: Roma - RM - 1999 - 145653 - Codice Fiscale e Partita I.V.A. 05780911003

ALLEGATO 4

Dipartimento di Emergenza Accettazione - Unità Operativa di Neurorianimazione

CONSENSO INFORMATO (1)

Io sottoscritto (specificare il grado di parentela)______________________________________

dichiaro di avere ricevuto dal Dottor (2) ______________________________________

esaurienti e dettagliate spiegazioni in merito alla proposta di utilizzare a scopo terapeutico la terapia denominata "mobilizzazione precoce" utilizzando l'apparecchiatura ERIGO HOCOMA.
Tale trattamento prevede la mobilizzazione precoce passiva ed attiva mediante un sistema robotizzato, che permetterebbe di ridurre le complicanze neurologiche connesse alla immobilità determinata dalle mie condizioni cliniche.

I rischi connessi a tale procedura consistono in fasi di ipotensione che, con adeguato monitoraggio ed opportuno controllo farmacologico, possono essere bene controllate e/o prevenute.

Dichiaro altresì di avere potuto discutere tutte le spiegazioni in merito alla sua applicazione , di porre tutte le domande che ho ritenuto necessarie e di avere ricevuto risposte soddisfacenti, come pure di avere avuto la possibilità di informarmi in merito ai particolari con persona di mia fiducia.

Accetto dunque liberamente di essere sottoposto al trattamento in oggetto, avendo compreso i rischi e i benefici che sono implicati.

Data __________ Firma del Medico che ha informato il paziente __________________

Data __________ Firma del paziente __________________

[*Nel caso il paziente non possa leggere e/o firmare*] (3)

Io sottoscritto______________________________________

Testimonio che il Dottor______________________________________

Ha esaurientemente spiegato al Sig.______________________________________

Le caratteristiche del trattamento in oggetto, e che lo stesso, avendo avuto la possibilità di fare tutte le domande che ha ritenuto necessarie, ha accettato liberamente di aderire.

Data __________ Firma del testimone indipendente __________________

---

1. Tale dichiarazione di consenso, deve essere firmata e datata personalmente dal paziente o dalle persona che ha condotto la discussione relativa al consenso informato.
2. Indicare il nome del medico che ha informato il paziente.
3. Se il paziente non è in grado di leggere e/o di firmare, un testimone indipendente dallo sperimentatore deve essere presente durante l'intera discussione relativa al consenso informato. Il testimone deve firmare e datare personalmente il consenso informato dopo che il modulo stesso e qualsiasi altra informazione scritta siano stati letti e spiegati al soggetto e questi abbia espresso il consenso verbale alla partecipazione allo studio.

OSPEDALE GENERALE DI ZONA "MORIGGIA – PELASCINI"
Classificato ai sensi Art. 1 Legge n. 132 del 12 Febbraio 1968 con D.R. n. 6880 del 12 Febbraio 1974
22015 **GRAVEDONA ED UNITI (CO)** - ☎ (0344) 92.111 - FAX (0344) 85.896
UFFICIO PRENOTAZIONI ☎ (0344) 92.401
ITALIA HOSPITAL S.p.A.
Capitale Sociale Euro 1.033.000,00 i.v. - Sede Legale: Corso Vittorio Emanuele II, 87 - 00186 ROMA
Ufficio Registro Imprese: Roma - RM - 1999 - 145653 - Codice Fiscale e Partita I.V.A. 05780911003

ALLEGATO 6

SCHEDA INFORMATIVA PAZIENTE

Unità Operativa/Dipartimento

....

Titolo della Sperimentazione e Numero di Protocollo

....

Gentile Signora/e,

in questa U.O. .... intendiamo svolgere una ricerca medico scientifica che si propone, attraverso uno studio pilota, di applicare precocemente un presidio robotizzato per una mobilizzazione precoce , allo scopo di ridurre le complicanze legate alla presenza di una grave cerebro lesione acquisita (GCLA) .

Per svolgere questa ricerca abbiamo bisogno della collaborazione e della disponibilità di pazienti affetti da GCLA, come Lei, che soddisfino i requisiti scientifici idonei allo studio.

Il personale è disponibile a spiegare con linguaggio adeguato le motivazioni dello studio e, qualora le sue condizioni cliniche non lo permettessero, a spiegarle ad una persona preposta .
Lo sperimentatore si impegna a tenere conto di eventuali sue volontà espresse consapevolmente, anche riferite da persona preposta.

Vogliamo altresì informarla che, anche se decidesse di non partecipare allo studio, Lei riceverà comunque tutte le terapie previste per la sua patologia, ed i medici continueranno a seguirla con la più completa attenzione assistenziale.

La preghiamo di leggere con attenzione quanto segue.

Un paziente affetto da Grave Cerebrolesione Acquisita (GCLA), necessita di un trattamento per la fase acuta, ed un trattamento per la fase post acuta di tipo riabilitativo. Vi è una forte evidenza clinica che la precocità di inizio del trattamento neuro riabilitativo, porti ad un significativo miglioramento, sia in termini di qualità che di tempi più brevi, nel recupero neuromotorio del paziente.

Questo studio si propone di valutare in termini di " fattibilità e sicurezza " , l'applicazione di tecniche neuro riabilitative in fase molto precoce, quando ancora il paziente è ricoverato in area intensiva, ma ha raggiunto una sufficiente stabilità clinica pur essendo ancora nella fase acuta della malattia.
La moderna tecnologia ci fornisce lo strumento adatto per potere fare ciò, un mobilizzatore computerizzato, che permette di verticalizzare il paziente e di programmare un cammino a carichi predeterminati dal terapeuta.

I rischi aggiuntivi attesi connessi all'applicazione precoce di questo trattamento, sono minimi. Il paziente, durante le fasi di trattamento, sarà sottoposto ad un monitoraggio esteso in continuo, ed ogni evento avverso, quale un calo della pressione arteriosa o eventi cardiologici aritmici, potranno essere immediatamente trattati e, in caso di persistenza, il trattamento neuroriabilitativo sarà sospeso.

Sistema Sanitario Regione Lombardia

In ogni caso, per eventuali danni direttamente o indirettamente derivanti dalla sperimentazione , l'Azienda Ospedaliera ha provveduto a stipulare per il paziente una polizza assicurativa, in ottemperanza al disposto dell'articolo 3 punto " f " del Decreto legislativo 24 Giugno 2003 n. 211.

Lo studio potrà in qualsiasi momento essere interrotto sia per richiesta del paziente o sua persona preposta, sia per volere del Medico. In questo caso, lo stesso dovrà fornire adeguata motivazione.

Ai sensi del decreto legislativo 196 del 30 Giugno 2003 " Codice in materia di protezione dei dati personali " , i dati personali del paziente verranno raccolti ed archiviati elettronicamente e saranno utilizzati esclusivamente per scopi di ricerca scientifica.
Il paziente ha il diritto di conoscere quali informazioni saranno memorizzate. L'accesso a tali dati sarà protetto dallo sperimentatore, nella persona dell'investigatore principale.

Per ulteriori informazioni e comunicazioni anche durante la sperimentazione, l'investigatore principale sarà sempre a disposizione .

Il protocollo è stato redatto in conformità alle Norme di Buona pratica Clinica ed è stato approvato dal Comitato Etico di………………………….. in data………………………………

COPIA
originale inviato il 10.12.2012 con Posta ordinaria (come da indica: zioni Ministero)

GRAVEDONA ED UNITI, 07/12/2012

Ministero della Salute
Direzione Generale farmaci e dispositivi medici
Ufficio VI - Indagini cliniche su dispositivi medici
Via Giorgio Ribotta, 5 - 00144 Roma

OGGETTO: DM-SC-ICPOM - Comunicazione di avvio di indagine clinica post market con Titolo 'La verticalizzazione precoce, attiva e passiva, mediante l#utilizzo dell#apparecchiatura ERIGO HOCOMA, nei pazienti affetti da GCLA (grave cerebro lesione acquisita) in Neurorianimazione: studio pilota di sicurezza e fattibilità.' e Codice di Protocollo Clinico '001'.

Ai sensi del Decreto Legislativo 46/97, art. 14, comma 8 come modificato dal Decreto Legislativo 37/10, si trasmette la comunicazione di avvio indagine clinica post-market, con dispositivo medico marcato CE ed utilizzato secondo la destinazione d'uso prevista e valutata ai fini della marcatura CE.
Di seguito sono riportati i principali dati identificativi dell'indagine clinica.

DATI RESPONSABILE DELLA COMUNICAZIONE

Denominazione: ITALIA HOSPITAL S.P.A. OSPEDALE MORIGGIA PELASCINI
Indirizzo: VIA PELASCINI, 3 **CAP/Zip Code:** 22015
Città: GRAVEDONA ED UNITI **Nazione:** ITALIA
Codice Fiscale: 05780911003 **Partita IVA/VAT Number:**
Indirizzo PEC (Posta Elettronica Certificata):
ospedaledigravedona@pec.gruppoitalcliniche.it

Persona di contatto

Nome: CARLA **Cognome:** NANNI
Email: soldarelli.moriggia@tiscali.it
Fax: **Telefono:**

DATI PROMOTORE

Soggetto Promotore: Altro soggetto Sponsor/Promotore
Denominazione: ITALIA HOSPITAL S.P.A. OSPEDALE MORIGGIA PELASCINI
Indirizzo: VIA PELASCINI, 3 **CAP/Zip Code:** 22015
Città: GRAVEDONA ED UNITI **Nazione:** ITALIA
Codice Fiscale/Partita IVA: 05780911003

Persona di contatto del Promotore

Nome: CARLA **Cognome:** NANNI
Fax: **Telefono:**
Email: soldarelli.moriggia@tiscali.it

CENTRI CLINICI IN CUI SI SVOLGE L'INDAGINE CLINICA

TIPOLOGIA STRUTTURA: STRUTTURA DI RICOVERO E CURA
Codice Struttura: 030031
Denominazione Struttura: OSPEDALE GENERALE DI ZONA "MORIGGIA-PELASCINI"
Denominazione Unità Organizzativa: TERAPIA INTENSIVA NEUROCHIRURGICA
Indirizzo: VIA PELASCINI, 3
CAP/Zip Code: 22015 **Città:** GRAVEDONA ED UNITI
Regione: LOMBARDIA **Centro coordinatore:** NO
Dati Sperimentatore
Nome: ROBERTO **Cognome:** VALSECCHI

Telefono: 0344-92715

Fax:

Dati Comitato etico

Denominazione: C.E. DELLA ASL DELLA PROVINCIA DI COMO

Data Parere Favorevole: 11/10/2012

DISPOSITIVI OGGETTO DI STUDIO

Banca Dati

Numero Banca Dati:

Dispositivo di confronto: NO

Dati del Fabbricante

Denominazione: HOCOMA AG

Nazione: SVIZZERA

Città: VOLKETSWIL

Indirizzo: INDUSTRIESTRASSE, 4

CAP/Zip Code: 8604

Codice Fiscale:

Dati del Mandatario

Denominazione: A CIRCLE S.P.A.

Nazione: ITALIA

Città: SAN PIETRO IN CASALE (BO)

Indirizzo: VIA FERRARA, 21

CAP/Zip Code: 40018

Codice Fiscale: 02431141205

Informazioni generali

Denominazione commerciale: ERIGO

Modelli:

Impiantabile attivo: NO

Classe di rischio:

Categoria dispositivo: APPARECCHIATURE SANITARIE E RELATIVI COMPONENTI ACCESSORI E MATERIALI

Gruppo dispositivo: STRUMENTAZIONE PER ESPLORAZIONI FUNZIONALI ED INTERVENTI TERAPEUTICI

Campo di applicazione: Medicina fisica e riabilitazione

DATI INDAGINE CLINICA

Informazioni sul disegno di studio

Tipologia di studio: Monocentrico (Nazionale)

Studio Osservazionale: NO

Tipo Controllo: Non Controllato

Tipologia di Confronto:

Randomizzazione: Non randomizzato

Denominazione Studio: La verticalizzazione precoce, attiva e passiva, mediante l#utilizzo dell#apparecchiatura ERIGO HOCOMA, nei pazienti affetti da GCLA (grave cerebro lesione acquisita) in Neurorianimazione: studio pilota di sicurezza e fattibilità.

CRO (Clinical Research Organization) coinvolta

Denominazione ed Indirizzo CRO:

Informazioni generali

Codice protocollo clinico: 001

Titolo del protocollo clinico: La verticalizzazione precoce, attiva e passiva, mediante l#utilizzo dell#apparecchiatura ERIGO HOCOMA, nei pazienti affetti da GCLA (grave cerebro lesione acquisita) in Neurorianimazione: studio pilota di sicurezza e fattibilità.

Obiettivo Dell'Indagine Clinica: Obbiettivo primario: # L#obiettivo del presente studio è verificare la fattibilità della procedura in assoluta sicurezza per il paziente. Obiettivi secondari: # Valutare gli effetti della verticalizzazione precoce, attiva e passiva, con sistema Erigo Hocoma, nei due gruppi di pazienti affetti da GCLA secondaria a traumi cranici e accidenti di natura vascolare con grave compromissione dello stato di coscienza. La valutazione di tali effetti sarà effettuata su :  Parametri clinici : valutazione neurologica mediante Coma Recovery Scale e scala di Ashworth.  Parametri strumentali : tutti gli acquisiti.

Numero di soggetti che si prevede di arruolare in Italia: 5

Numero di soggetti che si prevede di arruolare in totale: 5

Data di inizio sperimentazione: 17/12/2012

Data presunta di fine sperimentazione: 31/12/2013

Durata Follow Up: 6 Mesi

Popolazione di studio

Genere: Entrambi

Età: Da 18 ai 65 anni, Maggiori di 65 anni

Gravidanza/Allattamento:

Ambito Di Reclutamento: Pazienti ricoverati, Pazienti in emergenza

Le modifiche ed integrazioni dei dati, ivi comprese la data di interruzione e conclusione dell'indagine clinica, saranno tempestivamente comunicate al Ministero della Salute con riferimento al Codice di Protocollo Clinico sopra indicato.

Il/La sottoscritto/a NANNI CARLA, in qualità di persona autorizzata alla comunicazione dei dati relativi all'indagine clinica post market sopra descritta, consapevole delle sanzioni previste in caso di dichiarazioni false e mendaci (art. 76 del Testo Unico, D.P.R. 28 dicembre 2000,n.445), dichiara che i dati contenuti nel presente modulo sono esatti.

Il/La sottosctritto/a NANNI CARLA autorizza altresì il trattamento elettronico e la conservazione di tutti i dati compresi quelli personali presenti in questo modulo ai sensi e per gli effetti del D.lgs 196/2003.

Data **07/12/2012**

Firma ______

LE GEN. DI ZONA ITALIA HOSPITAL S.p.a. Moriggia-Pelascini, Gravedona ed Uniti (CO)

EFFICACIA DI UNA PRECOCE VERTICALIZZAZIONE IN TERAPIA INTENSIVA SULL'OUTCOME FUNZIONALE DEL PAZIENTE CON GCLA.

Zivi I, Valsecchi R, Bonini S, Zarucchi A, Molatore K, Maffia S, Frazzitta G.

Introduzione

L'outcome dei pazienti con alterazione dello stato di coscienza secondaria a grave cerebrolesione acquisita (GCLA) sembra essere positivamente correlato con la precocità dell'attivazione del percorso neuroriabilitativo; tuttavia, l'opportunità di una precoce ed intensiva mobilizzazione sin dalle prime fasi dell'ospedalizzazione è tuttora oggetto di discussione in letteratura.

Una delle fasi fondamentali nella riabilitazione del grave cerebroleso è rappresentata dalla verticalizzazione, sia per la ricchezza di informazioni sensitive e sensoriali che la stazione eretta fa afferire al cervello, sia perché favorisce una migliore circolazione ematica e liquorale cerebrale rispetto alla posizione clinostatica a letto.

L'Erigo® è un tilt table dotato di un dispositivo robotico in grado di determinare un movimento steppante passivo degli arti inferiori del paziente. Il suo vantaggio, rispetto ai normali tilt table, è rappresentato dall'attivazione della pompa muscolare che, impedendo la stasi venosa declive ed aumentando il preload cardiaco, riduce le reazioni ipotensive ortostatiche.

Il nostro gruppo ha già dimostrato in uno studio pilota la fattibilità e la sicurezza della verticalizzazione precoce (nel primo mese dall'evento acuto) di pazienti con grave cerebrolesione acquisita, eseguita in terapia intensiva tramite "Erigo"® *(J Head Trauma Rehabil. 2015 Jul-Aug;30(4):290-2*).

Scopo dello studio

Valutare se la verticalizzazione precoce con Erigo in terapia intensiva ha effetto sull'outcome funzionale a breve e lungo termine dei pazienti affetti da GCLA.

Metodi

Saranno arruolati 40 pazienti ricoverati consecutivamente presso la nostra terapia intensiva a ≤24 ore da un evento traumatico o vascolare responsabile di una severa e prolungata alterazione della coscienza e successivamente trasferiti presso il reparto di neuroriabilitazione.

Criteri di inclusione: età 18-75, diagnosi di coma, stato vegetativo o stato di minima coscienza in terza giornata dall'evento.

Criteri di esclusione: instabilità emodinamica non controllabile con amine, instabilità della pressione intracranica, sepsi, controindicazioni ortopediche alla verticalizzazione, trombosi venosa profonda, peso>130 kg, altezza >210 cm.

Tutti i pazienti saranno sottoposti a valutazione oggettiva mediante le seguenti scale: Glasgow Coma Scale, Disability Rating Scale, Levels of Congitive Functioning e Coma Recovery Scale revisited.

20 pazienti saranno sottoposti a quindici sessioni (5/settimana per 3 settimane) di graduale verticalizzazione tramite Erigo in terapia intensiva. Durante ogni seduta verranno continuativamente monitorati e registrati pressione arteriosa (PA), frequenza cardiaca (FC), e saturimetria (SpO2).

20 pazienti durante la degenza in terapia intensiva riceveranno trattamento fisioterapico standard di mobilizzazione a letto.

Tutti i pazienti, al termine del percorso di cure intensive, verranno trasferiti in neuroriabilitazione, ove proseguiranno con il programma riabilitativo standard.

Le scale di valutazione utilizzate all'ingresso in terapia intensiva verranno sottoposte anche all'ingresso ed alla dimissione dalla riabilitazione.
Verranno inoltre registrati in tutti i pazienti i tempi si svezzamento dai presidi (CVC, CV, PEG, tracheocannula).

I punteggi ottenuti alle scale ed i tempi di svezzamento dai presidi verranno quindi confrontati tra i due gruppi (Erigo si/Erigo no), allo scopo di definire se il trattamento con Erigo consenta di migliorare l'outcome neurologico e funzionale dei pazienti con GCLA rispetto al trattamento standard.
