# Supplementary material for: Effectiveness of a Very Early Stepping Verticalization Protocol in Severe Acquired Brain Injured Patients: A Randomized Pilot Study in ICU
Source: PLoS One. 2016 Jul 22;11(7):e0158030. doi: 10.1371/journal.pone.0158030 (PMC4957764; doi:10.1371/journal.pone.0158030)
Supplement: S2 Protocol — (DOCX) [file pone.0158030.s004.docx]

**Study Protocol:**

**EFFECTIVENESS OF A VERY EARLY STEPPING VERTICALIZATION IN SEVERE BRAIN INJURIED PATIENTS**

G. Frazzitta, I. Zivi, R. Valsecchi, S. Bonini, S. Maffia, K. Molatore, L. Sebastianelli, A. Zarucchi, D. Matteri, G. Ercoli, R. Maestri, L. Saltuari

**Aim of the study**

Define if a very early stepping verticalization in ICU is effective on short-term and long-term outcome of patients with vegetative state or minimally conscious state after severe acquired brain injury.

**Study design**

Type of study: RCT

Location: Intensive Care Unit rooms (‘Moriggia-Pelascini’ Hospital, Gravedona ed Uniti –CO-, Italy)

Personnel: Intensive Care team (anesthesiologists and nurses) + Neurorehabilitation team (Neurologist, Physiatrist and Physiotherapists)

**Population**

40 consecutive ICU patients admitted within 24 hours from a severe ABI.

Written informed consent (next of kin).

Inclusion criteria:

- age ≥18 years
- Glasgow Coma Scale ≤8 for ≥24h from the event
- diagnosis of VS or MCS (revisited Coma Recovery Scale) on the third day after injury
- adequate pulmonary gas exchanging function (arterial O2 pressure/O2 flux ratio ≥250)
- stable hemodynamics (even if obtained with amines support)

Exclusion criteria:

- sedation
- unstable intracranial pressure
- cerebral perfusion pressure <60 mmHg
- fractures or skin lesions (thorax, abdomen, lower limbs)
- deep vein thrombosis
- body weight >130 kg, height >210 cm

Randomization: (block randomization procedure)

- *Experimental group*: early stepping verticalization protocol
- *Control group*

**Protocol**

- Experimental group: 50% patients
  - 15 Verticalization sessions with Erigo
    - Start: between the 3^rd^ and the 30^th^ day after injury
    - Frequency: one session per day; 5 sessions per week (Mon-Fri); 3 consecutive weeks
    - Session duration: 30 minutes
    - Verticalization phases:
      - Gradual increase of the slope of the tilt table: from 0° to 20°, 40° and then 60° in 9 minutes.
      - Verticalization at 60° for 20 minutes
      - Gradual decrease of the slope of the tilt table from 60° to 0° in 1 minute
    - Stepping frequency: 20 steps/min
    - Continuous monitoring: heart rate, mean arterial pressure, oxygen saturation
    - Supervision: one physiotherapist
    - Personnel available during sessions: ICU nurse and anesthesiologist
    - Criteria for session interruption
      - mean arterial pressure ≤70 mmHg
      - heart rate ≤40 or ≥150 bpm
      - oxigen saturation ≤90%
      - traumatic dislodgement of a device (tracheal cannula, venous or arterial catheter, bladder catheter).
    - Criteria for withdrawal from the study: neurological worsening or myocardial infarction
  - Conventional physiotherapy: 50% patients
    - 60 minutes a day (Mon-Fri) before starting verticalization sessions
    - 30 minutes a day (Mon-Fri) during verticalization period
- Control group:
  - conventional physiotherapy for 60 minutes a day (Mon-Fri) throughout the ICU stay
- Both groups:
  - Moving to Neurorehabilitation: after clinical stabilization and completion of the protocol
  - 6-month individualized rehabilitative program

**Data collection**

- Descriptive: age, sex, etiology, comorbidities, side of the main brain damage
- Outcome measures:
  - Glasgow Coma Scale, Disability Rating Scale, Coma Recovery Scale revised, Levels of Cognitive Functioning
  - Scores assessed from a blinded investigator at
    - T0: 3^rd^ day from the injury
    - T1: ICU discharge
    - T2: Rehabilitation discharge
  - Short-term outcome: differences of the scores between T1 and T0
  - Long-term outcome: differences of the scores between T2 and T0

**Statistical analysis**

- Descriptive statistics: mean±SD or median (lower quartile, upper quartile)
- Outcome variables: non-parametric one-factor ANOVA
